# Analytic representative element rate decline models for naturally fractured reservoir depletion

**DOI:** 10.1038/s41598-024-59023-5

**Published:** 2024-04-16

**Authors:** R. D. Hazlett, T. Syrymov, R. Younis

**Affiliations:** 1 School of Mining and Geosciences, Nazarbayev University, Astana, Kazakhstan; 2 Harold Vance Department of Petroleum Engineering, Texas A&M University, College Station, USA

**Keywords:** Fracture, Transfer, Multiscale, Analytical, Diffusivity, Transients, Structural geology, Sedimentology, Hydrology, Engineering, Materials science

## Abstract

Representative single anisotropic matrix block 2D Green’s function models for depletion through fully-penetrating, vertical fractures through different numbers of fracture faces are constructed that analytically capture both fracture and block depletion with fracture-matrix mass transfer. The 1D Green’s function for a fracture system is likewise solved in terms of the time evolution of average fracture pressure. While transient average pressure values are not inherently measurable, they are transformed into cumulative production or instantaneous flowrate values, thus producing new rate decline model functional forms. Primary variables in assembling the interacting systems model are the volume ratio, *V*_*f*_* /V*_*m*_, permeability ratio, *k*_*f*_* /k*_*x*_, and geometry, (*a/b*)(*k*_*y*_*/k*_*x*_), with the last term accounting for both block shape and permeability anisotropy. We construct interacting systems models in terms of various ratios of *V*_*f*_* /V*_*m*_, and *k*_*f*_* /k*_*x*_ for three fracture architecture prototypes: representative matrix blocks depleted by 4, 2, or 1 contacting fractures. The single matrix block models can be migrated to ones for heterogeneous systems using superposition and matrix block distributions, as demonstrated with a binary distribution of block sizes with variable fractions. Analytic solutions for rate decline problems can be used to understand the production signatures of naturally fractured reservoirs and interpretation of fracture volume fraction, permeability ratio, average matrix block size, and measures of heterogeneity.

## Introduction

The Earth’s upper crust is characterized by common structural features, such as fractures. Most oil and gas reservoirs are naturally fractured to some degree. Moreover, about 60 percent of the known oil and gas reserves throughout the world are characterized as belonging to fractured fields^1^. There are numerous models to characterize naturally fractured reservoir behaviour. One of the most famous, but perhaps oversimplified, models is the dual-porosity model. Application of this model requires the problematic estimation of fracture volume fraction. Generally, it is considered that fractures have very high permeability but low storage capacity, therefore very low porosity.

There has been a significant increase in the study of fluid dynamics and transport mechanisms in fractured, porous environments in the past half-century with significant progress. The works of many authors gave insight into how flow occurs and transport processes that take place in naturally fractured reservoirs. However, conceptual, and mathematical issues still exist. There are several reasons fractured porous media does not have a suitable model. First, naturally fractured reservoirs are extremely heterogeneous, and therefore, it is not clear how matrix-fracture systems communicate. Second, because of the complexity of the system, it is difficult to conceptualize and understand the flow of fluids and transport. Finally, there is a lack of data to build a reliable deterministic or statistical mathematical three-dimensional field fracture model that accounts for fracture flow and storage behaviour at all scales at which fractures are observed.

Barenblatt et al.^2^ proposed to divide fractured formations into interacting systems: high permeability, low porosity fractures along with low permeability, high porosity matrix. These two systems communicate using an exchange function—The matrix-fracture transfer function. The reservoir description and the appropriateness of the matrix-fracture transfer function fundamentally impact the quality of fractured reservoir modelling. The transfer function portrays the physics of pore-scale phenomena along with the scale-up processes, enabling more efficient coarse scale modelling. Early models simplified the reservoir system to a representative matrix block surrounded by fractures. This model is simple and does not require significant computational demand, as does the discrete-fracture approach. The dual-continuum approach represents a fractured medium by a representative elementary volume. One of the simplest models representing the dual continuum approach is the “dual porosity” model, which has existed in the literature for a considerable time^3^. In this model, authors consider the fracture system as the continuous medium (a continuum) and matrix as a set of discrete porous blocks embedded in the fracture continuum. An extension to the dual-porosity approach, called “dual permeability”, was advocated by Gerke and van Genuchten^4^ in hydrology and Blaskovich et al.^5^ and Hill and Thomas^6^ in hydrocarbon reservoir simulation. Additionally, advances were reviewed by Chen^7^. Extension include communication between matrix elements—not only between matrix and fracture. These model conceptualizations remain the heart of many present-day fractured reservoir simulators.

There was significant early progress in developing numerical solution methodology for complex fracture systems in reservoir simulation^3,8,9^. Simple models like “dual-porosity” are computationally efficient, but they are inappropriate for complex systems where multi-scale phenomena dominate in space and time. Numerical models have been proposed to capture the effect of contributing physics on multiple scales. Researchers have conducted many experiments with different approaches to understand physics at different scales^10,11^.

Authors have suggested transfer functions to understand the interaction between matrix and fracture domains^2,12–15^. They proposed another way of dealing with the transfer function. Specifically, it was necessary to find a solution that represents the behaviour of average pressure and saturation within a grid block after a disturbance at the boundary, thus avoiding the need for spatial resolution. This approach looks very useful and attractive to numerical simulation, because numerical simulation works with average properties ascribed to cells with no spatial detail below the resolution of a cell. The question about such transfer functions is if they can adequately represent subscale physics. Thus, grid blocks deplete and empty into the fracture system, while average properties allow for the conservation of mass.

Researchers have experimented with different approaches to capture the effects of contributing physics at different scales. Among these include Embedded Discrete Fracture Models (EDFM) and Multiple INteracting Continua (MINC) or nested layers in gridding^16^. Bosma et al.^17^ presented a multiscale method for discrete fracture modelling using unstructured grids where grids at one scale feed information to another that conforms to fracture geometry. Xu and Sepehrnoori^18^ developed an embedded discrete fracture model that takes advantage of corner point grids with the advantage of compatibility with commercial simulators.

In attempts to address inherent heterogeneity, Maier and Geiger^19^ demonstrated multi-rate dual porosity models that introduce a distribution of transfer functions per block to capture more variability in properties, including the effects of smaller-scale fractures, in conjunction with a discrete fracture model that captures the largest fracture sets. Belani and Jalali^20^ examined pseudo-steady state and transient model behaviour for uniform and bimodal block size distributions but employed solutions with radial symmetry and an infinite acting boundary condition. Interestingly, these authors noted that a closed form analytical solution was unavailable to describe transient flow. Contrary to the paper title, theirs is a forward model for prescribed block size distribution. Gong and Rossen^1^ tried to reduce dual permeability fractured reservoir simulation to the determination of shape factors (transfer functions) on only the dominant fluid-carrying fracture subset. Amiry^21^ gave an insightful summary of state-of-the-art naturally fractured reservoir modelling and carefully proposed a method within computational reach for real world applications that captured subscale information in matrix blocks. He proposed scaled matrix recovery curves to prescribe transfer functions coming from experiments or single block simulations.

## Theory

### Matrix depletion model development

#### Four-sided drainage

Hazlett and Younis^22^ presented a transient baseline solution for depletion of single matrix blocks and effective steady state transport properties for matrix blocks with embedded fractures. Following that work, the solution to the Green’s function for depletion from a matrix block in a formation of thickness *h* with area 4*ab* and directional permeabilities *k*_*x*_ and *k*_*y*_ into a surrounding constant pressure fracture system is

1 $$\frac{{P}_{m}\left(x,y;{t}_{D}\right)}{{P}_{i}}=\frac{16}{{\pi }^{2}}\sum_{l,n=0}^{\infty }\frac{{\left(-1\right)}^{l+n}}{\left(2l+1\right)\left(2n+1\right)}{\text{cos}}\left[\frac{\pi \left(2l+1\right)x}{2a}\right]{\text{cos}}\left[\frac{\pi \left(2n+1\right)y}{2b}\right]{e}^{-\frac{{\pi }^{2}}{4}\left[\frac{{k}_{x}{\left(2l+1\right)}^{2}}{{a}^{2}}+\frac{{k}_{y}{\left(2n+1\right)}^{2}}{{b}^{2}}\right]\tau }$$

**Figure 1:**
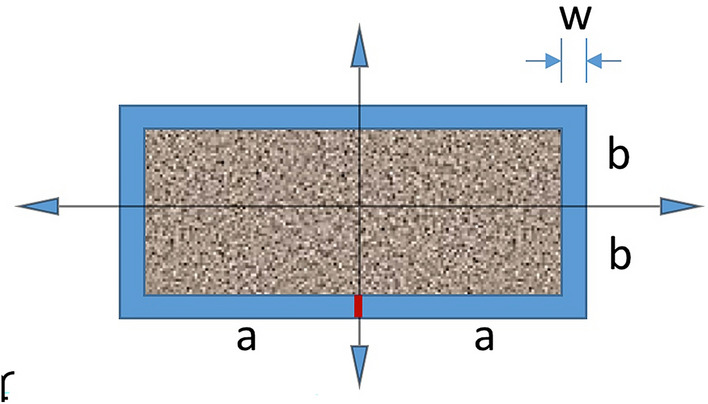
Interacting systems model for matrix depletion in surrounding fully-penetrating vertical fractures. In the matrix, P_m_(x, y; 0) = 1, P_m_(± a, ± b; t = 0^+^) = 0. In the surrounding set of 1D fractures of length 2(a + b) with a withdrawal point at (0,−b), in terms of arclength, s, P_f_ (s, 0) = 1, P_f_ (0, t = 0^+^) = 0.

Here, *P* is pressure at arbitrary observation point (*x*, *y*) with the subscripts *m* and *i* representing matrix and initial values with dimensionless time, $\tau = t/(\mu c)$, with *µ* as viscosity, *c* as compressibility of only the fluid, and *t* as dimensioned time. We omit the porosity from the definition and do not include the total compressibility. Note this is a double infinite summation on the dummy variables *l* and *n* to capture all reflections from the method of images necessary to give rise to the constant pressure boundary condition imposed on the given matrix boundaries at ± *a* and ± *b*. A depiction of the model geometry is given in Fig. 1, where we see a 2D matrix block in plan view of thickness, *h*, and dimensions 2*a x* 2*b* surrounded by a fracture of average aperture, *w*. This is a depiction of primary depletion for matrix blocks “bleeding” into contacting fractures belonging to a high permeability gathering system. As a solution for Dirichlet boundaries, once material reaches the surrounding fracture, it is removed from the system.

The pressure in the depleting fracture, as a function of distance *s* from a designated withdrawal point, is given as *P*_*f*_ (*s*). We analyse the depletion of fracture and matrix independently to understand the corresponding time scales. The matrix can contain subscale fractures; however, initially, it is assumed that the microstructure fabric, including subscale fractures, can be captured by effective directional permeabilities. While Hazlett and Younis^22^ analysed how and where matrix fluids exit into fractures to understand spatial anisotropy in the matrix-fracture transfer function, we are reminded that material balance is an expression of single cell simulation and is sufficient to capture total fluid expelled, provided no spatial information is needed.

The cell average pressure in the matrix, ${\overline{{\text{P}}} }_{m}\left(t\right)$, is connected to cumulative recovery through material balance, which Hazlett and Younis^22^ give relative to the initial pressure, *P*_*i*_, as

2 $$\frac{{\overline{{\text{P}}} }_{m}\left(t\right)}{{{\text{P}}}_{{\text{i}}}}=\frac{64}{{\pi }^{4}}\sum_{l,n=0}^{\infty }\frac{{\text{exp}}\left[-\frac{{\pi }^{2}}{4}\left({\left(2l+1\right)}^{2}+\left(\frac{{k}_{y}}{{k}_{x}}\right){\left(\frac{a}{b}\right)}^{2}{\left(2n+1\right)}^{2}\right)\left(\frac{{k}_{x}\tau } q = - c \cdot V_{{ref}} \frac{{d\bar{p}}}{{dt}} {{a}^{2}}\right)\right]}{{\left(2l+1\right)}^{2}{\left(2n+1\right)}^{2}}$$

The time derivative, allowing access to instantaneous flow rate, follows as

3 $$\frac{1}{{{\text{P}}}_{{\text{i}}}}\frac{{\text{d}}{\overline{{\text{P}}} }_{m}\left(t\right)}{\mathrm{d\tau }}=-\frac{16}{{\pi }^{2}}\left(\frac{{k}_{x}}{{a}^{2}}\right)\sum_{l,n=0}^{\infty }\left[{\left(2n+1\right)}^{-2}+\left(\frac{{k}_{y}}{{k}_{x}}\right){\left(\frac{a}{b}\right)}^{2}{\left(2l+1\right)}^{-2}\right]\mathrm{ exp}\left[-\frac{{\pi }^{2}}{4}\left({\left(2l+1\right)}^{2}+\left(\frac{{k}_{y}}{{k}_{x}}\right){\left(\frac{a}{b}\right)}^{2}{\left(2n+1\right)}^{2}\right)\left(\frac{{k}_{x}\tau }{{a}^{2}}\right)\right]$$

The Bourdet derivative is often used to diagnosis flow regime and is easily constructed analytically as

4 $$\frac{{{\text{d}}\overline{{\text{P}}} _{m} \left( {{\text{t}}_{D} } \right)}}{{{\text{dlnt}}_{D} }} = {\text{t}}_{D} \cdot \frac{{{\text{d}}\overline{{\text{P}}} _{m} \left( {{\text{t}}_{D} } \right)}}{{{\text{dt}}_{D} }}$$

where the dimensionless time, ${t}_{D}=\left(\frac{{k}_{x}}{{a}^{2}}\right)\tau$ , is introduced as a lumped dimensionless stretched time.

Physical measurement of the average pressure within a matrix block is not possible, so we relate average pressure and cumulative and instantaneous flow rate for either matrix or fracture as

5 $$q = - c \cdot V_{{ref}} \frac{{d\bar{p}}}{{dt}}$$

where the compressibility, *c*, is a property of the fluid only, identical in the matrix and fracture, and the reference volume, *V*_*ref*_, is the subsystem fluid volume in either case. Thus, the production from matrix with Dirichlet boundaries is

6 $${{\text{q}}}_{m,4}=\frac{{P}_{i}{V}_{m}}{\mu }\frac{16}{{\pi }^{2}}\left(\frac{{k}_{x}}{{a}^{2}}\right)\sum_{l,n=0}^{\infty }\left[{\left(2n+1\right)}^{-2}+\left(\frac{{k}_{y}}{{k}_{x}}\right){\left(\frac{a}{b}\right)}^{2}{\left(2l+1\right)}^{-2}\right]{\text{exp}}\left[-\frac{{\pi }^{2}}{4}\left({\left(2l+1\right)}^{2}+\left(\frac{{k}_{y}}{{k}_{x}}\right){\left(\frac{a}{b}\right)}^{2}{\left(2n+1\right)}^{2}\right){t}_{D}\right]$$

#### Two-sided drainage

**Figure 2:**
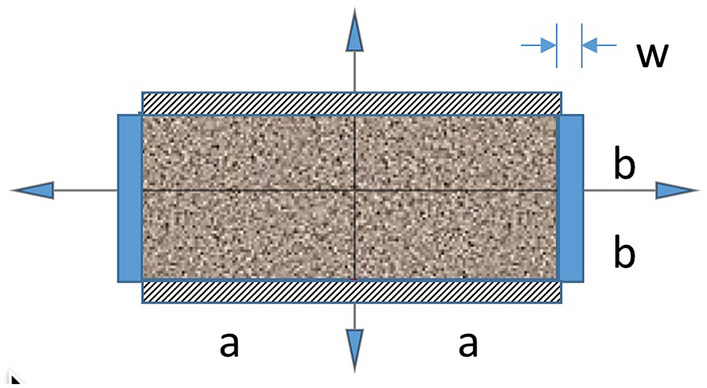
Interacting systems model for matrix depletion from parallel fully-penetrating vertical fractures. In the matrix, P_m_(x, y; 0) = 1, P_m_(± a, y; t = 0^+^) = 0 with Neumann boundaries at y =  ± b. In the two 1D fractures of length 2b with withdrawal points at (± a,−b), in terms of arclength, s, P_f_ (s; 0) = 1, P_f_ (0; t = 0^+^) = 0.

We add another depletion model for the case where matrix is drained from parallel fractures without cross-fracture sets. Other sides are considered sealed. This is depicted in Fig. 2.

For this model, flow becomes 1D with

7 $$\frac{{\overline{{\text{P}}} }_{m,2}\left(t\right)}{{{\text{P}}}_{{\text{i}}}}=\frac{4}{{\pi }^{2}}\sum_{l=0}^{\infty }\frac{{e}^{-\frac{{\pi }^{2}}{4}\left[\frac{{k}_{x}}{{a}^{2}}{\left(2l+1\right)}^{2}\right]\tau }}{{\left(2l+1\right)}^{2}}$$

so

8 $${{\text{q}}}_{m,2}=\frac{{P}_{i}{V}_{m}}{\mu }\left(\frac{{k}_{x}}{{a}^{2}}\right)\sum_{l=0}^{\infty }{e}^{-\frac{{\pi }^{2}}{4}\left[{\left(2l+1\right)}^{2}\right]{t}_{D}}$$

For fully-penetrating fractures, the dependence on thickness only enters through the total matrix volume.

#### One-sided drainage

**Figure 3:**
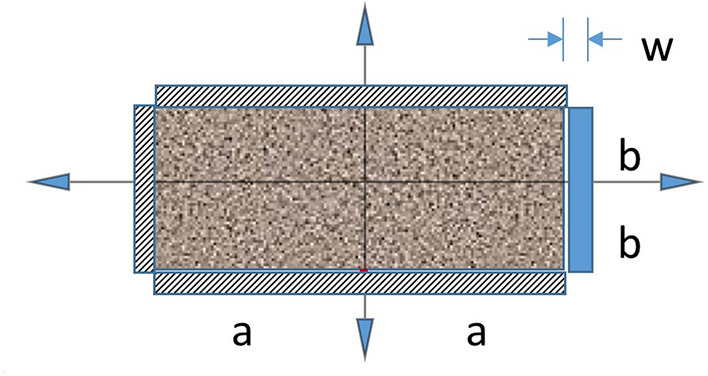
Interacting systems model for matrix depletion from a single fully-penetrating vertical fracture. In the matrix, P_m_(x, y; 0) = 1, P_m_(a, y; t = 0^+^) = 0 with Neumann boundaries at y =  ± b and x = −a. In the 1D fracture of length 2b with a withdrawal point at (a,-b), in terms of arclength, s, P_f_ (s; 0) = 1, P_f_ (0; t = 0^+^) = 0.

The last variation is an otherwise sealed matrix block with a fracture on only one side. Since symmetry is seen in Fig. 2, we have a no flow boundary at x = 0. Thus, the one-sided drainage problem, illustrated in Fig. 3, is contained within the parallel fracture drainage solution, yet for a different aspect ratio *a/b*. Yet, for 1D problems, the aspect ratio is irrelevant except when needing dimensioned flowrates.

For a revised coordinate system with origin moved to (-a, -b) that is insulated at x = 0 but kept at constant temperature, *θ*, at *x* = *L* and initial temperature distribution as *f(x)*, Carslaw and Jaeger^23^ give the solution to this 1-D problem for temperature, *T*, as

9 $$T = \theta + \frac{2}{L}\mathop \sum \limits_{n = 0}^{\infty } e^{{ - \frac{{\pi^{2} }}{4}\left[ {\frac{{k_{x} }}{{L^{2} }}\left( {2n + 1} \right)^{2} } \right]t}} cos\frac{{\left( {2n + 1} \right)\pi x}}{2L}\left\{ {\frac{{2L\left( { - 1} \right)^{n + 1} V}}{{\left( {2n + 1} \right)\pi }} + \mathop \smallint \limits_{0}^{L} f\left( x \right)cos\frac{{\left( {2n + 1} \right)\pi x}}{2L}dx} \right\}$$

For the problem definition such that *θ* = 0 and uniform initial temperature, i.e. *f(x)* = 1, we have

10 $$T=\frac{4}{\pi }\sum_{n=0}^{\infty }\left\{\frac{{\left(-1\right)}^{n}}{2n+1}\right\}{e}^{-\frac{{\pi }^{2}}{4}\left[\frac{{k}_{x}}{{L}^{2}}{\left(2n+1\right)}^{2}\right]\tau }cos\frac{\left(2n+1\right)\pi x}{2L}$$

For which the average becomes

11 $$\overline{T }=\frac{8}{{\pi }^{2}}\sum_{n=0}^{\infty }\frac{{e}^{-\frac{{\pi }^{2}}{4}\left[\frac{{k}_{x}}{{L}^{2}}{\left(2n+1\right)}^{2}\right]\tau }}{{\left(2n+1\right)}^{2}}$$

Making the analogy between pressure and temperature and maintaining consistency in geometry with *L* = *2a*, we obtain

12 $${q}_{m,1}=\frac{{P}_{i}{V}_{m}}{2\mu }\left(\frac{{k}_{x}}{{a}^{2}}\right)\sum_{n=0}^{\infty }{e}^{-\frac{{\pi }^{2}}{16}\left[{\left(2n+1\right)}^{2}\right]{t}_{D}}$$

Thus, the solutions for one and two-sided drainage are similar, but the one-sided matrix drainage will be slowed significantly due to the longer drainage path.

### Fracture depletion model development

To the development for matrix depletion into fractures, we must add the early depletion of the contacting fractures themselves. Depletion in the fracture is modeled as a 1D channel of width, *w*, with uniform initial pressure matching that of the matrix, followed by a sudden change in pressure at a specific withdrawal point. Alternative withdraw points are certainly possible, some of which may break the fracture into simultaneous depleting tributaries with different lengths, and thus volume. The observed pressure depletion in such a case would be a volume-weighted average pressure between contributing tributaries.

#### Four-sided drainage

For matrix blocks surrounded by fractures, we must choose the withdraw point in communication with the drainage system. For the case of matrix surrounded by fractures on four sides, this is indicated in red on Fig. 1 for simplicity. However, any point on the surrounding fracture would result in the same interpretation of two 1-D flow channels of equal length.

Carslaw & Jaeger^23^ give the 1-D solution for unit initial value on *-L* < *x* < *L* and boundaries maintained at zero as

13 $${P}_{f}\left(x,L,t\right)=\frac{4}{\pi }\sum_{l=0}^{\infty }\frac{{\left(-1\right)}^{l}}{2l+1}{e}^{-\frac{{k}_{f}{\pi }^{2}}{4{L}^{2}}{\left(2l+1\right)}^{2}\tau }{\text{cos}}\frac{\left(2l+1\right)\pi x}{2L}$$

where we introduce the subscript to denote properties of a fracture. Of course, the porosity in the fracture in the dimensionless time definition is taken as unity. Due to symmetry, the clockwise and counter-clockwise pressure pulses meet with total travel distance, *L* = (*a* + *b*), for the block surrounded by fractures and *L* = *b*, otherwise. If we also assume the cubic law permeability for flow in a slit, $k=\frac{{w}^{2}}{12}$, we can get an expression for average pressure in the fracture dependent only on geometry. However, the work of Mustafayev and Hazlett^24^ would indicate this is a poor realistic choice for fractures with natural roughness, so we leave the result in terms of *k*_*f*_. We note that the volume doubles for the two-fracture system over that of the single fracture. The time dependent average pressure is then in terms of the geometry of Fig. 1,

14 $$\frac{{\overline{{\text{P}}} }_{f,4}\left(t\right)}{{{\text{P}}}_{{\text{i}}}}=\frac{8}{{\pi }^{2}}\sum_{n=0}^{\infty }\frac{{e}^{-\frac{{\pi }^{2}{k}_{f}{\left(2n+1\right)}^{2}\tau }{4{\left(a+b\right)}^{2}}}}{{\left(2n+1\right)}^{2}}$$

yielding

15 $${{\text{q}}}_{f,4}=2\frac{{P}_{i}{V}_{m}}{\mu }\left(\frac{{V}_{f}}{{V}_{m}}\right)\left[\frac{1}{{\left(1+\frac{b}{a}\right)}^{2}}\right]\left(\frac{{k}_{f}}{{k}_{x}}\right)\left(\frac{{k}_{x}}{{a}^{2}}\right)\sum_{n=0}^{\infty }{e}^{-\frac{{\pi }^{2}{\left(2n+1\right)}^{2}}{4}\left[\frac{1}{{\left(1+\frac{b}{a}\right)}^{2}}\right]\left(\frac{{k}_{f}}{{k}_{x}}\right){t}_{D}}$$

#### One and two-sided drainage

In the fracture drainage consideration of Figs. 2,3, the problems are identical, provided both arms of in the 2-sided fracture drainage are connective to the overall collection system. We get

16 $$\frac{{\overline{{\text{P}}} }_{f,2}\left(t\right)}{{{\text{P}}}_{{\text{i}}}}=\frac{{\overline{{\text{P}}} }_{f,1}\left(t\right)}{{{\text{P}}}_{{\text{i}}}}=\frac{8}{{\pi }^{2}}\sum_{n=0}^{\infty }\frac{{e}^{-\frac{{\pi }^{2}{k}_{f}{\left(2n+1\right)}^{2}\tau }{4{b}^{2}}}}{{\left(2n+1\right)}^{2}}$$

and identical expressions for flowrate, given that the difference is incorporated in the fracture volume, *V*_*f*_.

17 $${\text{q}}_{{f,2}} = {\text{q}}_{{f,1}} = 2\frac{{P_{i} V_{m} }}{\mu }\left( {\frac{{V_{f} }}{{V_{m} }}} \right)\left( {\frac{a}{b}} \right)^{2} \left( {\frac{{k_{f} }}{{k_{x} }}} \right)\left( {\frac{{k_{x} }}{{a^{2} }}} \right)\sum\limits_{{n = 0}}^{\infty } {e^{{ - \frac{{\pi ^{2} }}{4}\left( {2n + 1} \right)^{2} \left( {\frac{a}{b}} \right)^{2} \left( {\frac{{k_{f} }}{{k_{x} }}} \right)t_{D} }} }$$

### Single block interacting fracture systems depletion model

Since the Green’s function solution for matrix depletion places a stationary pressure boundary condition for t = 0^+^, we can only add matrix and fracture solutions, treating them as independent events, provided the transients in the fracture are essentially complete prior to any appreciable production from the matrix. Otherwise, the pressure in the bounding fracture would remain a function of space and time. If the time scales are appreciably different, we can piece together the signature of a dual porosity system as

18 $${{\text{q}}}_{f,4}+{{\text{q}}}_{m,4}=\frac{{P}_{i}{V}_{m}}{\mu }\left(\frac{{k}_{x}}{{a}^{2}}\right)\left\{\begin{array}{c}\left(\frac{{V}_{f}}{{V}_{m}}\right)\left(\frac{{k}_{f}}{{k}_{x}}\right)\left[\frac{2}{{\left(1+\frac{b}{a}\right)}^{2}}\right]\sum\limits_{n=0}^{\infty }{e}^{-\frac{{\pi }^{2}}{4}{\left(2n+1\right)}^{2}\left[\frac{1}{{\left(1+\frac{b}{a}\right)}^{2}}\right]\left(\frac{{k}_{f}}{{k}_{x}}\right){t}_{D}}\\ +\frac{16}{{\pi }^{2}}\sum\limits_{l,m=0}^{\infty }\left[{\left(2m+1\right)}^{-2}+\left(\frac{{k}_{y}}{{k}_{x}}\right){\left(\frac{a}{b}\right)}^{2}{\left(2l+1\right)}^{-2}\right]{\text{exp}}\left[-\frac{{\pi }^{2}}{4}\left({\left(2l+1\right)}^{2}+\left(\frac{{k}_{y}}{{k}_{x}}\right){\left(\frac{a}{b}\right)}^{2}{\left(2m+1\right)}^{2}\right){t}_{D}\right]\end{array}\right\}$$

For the other two interacting model systems, we get more simplified results

19 $${\text{q}}_{2} = \frac{{P_{i} V_{m} }}{\mu }\left( {\frac{{k_{x} }}{{a^{2} }}} \right)\left\{ {2\left( {\frac{{V_{f} }}{{V_{m} }}} \right)\left( {\frac{a}{b}} \right)^{2} \left( {\frac{{k_{f} }}{{k_{x} }}} \right)\sum\limits_{{n = 0}}^{\infty } {e^{{ - \frac{{\pi ^{2} }}{4}\left( {2n + 1} \right)^{2} \left( {\frac{a}{b}} \right)^{2} \left( {\frac{{k_{f} }}{{k_{x} }}} \right)\left( {\frac{{k_{x} }}{{a^{2} }}} \right)t}} } + \sum\limits_{{l = 0}}^{\infty } {e^{{ - \frac{{\pi ^{2} }}{4}\left[ {\left( {2l + 1} \right)^{2} } \right]t_{D} }} } } \right\}$$

and

20 $${\text{q}}_{1} = \frac{{P_{i} V_{m} }}{\mu }\left( {\frac{{k_{x} }}{{a^{2} }}} \right)\left\{ {2\left( {\frac{{V_{f} }}{{V_{m} }}} \right)\left( {\frac{a}{b}} \right)^{2} \left( {\frac{{k_{f} }}{{k_{x} }}} \right)\sum\limits_{{n = 0}}^{\infty } {e^{{ - \frac{{\pi ^{2} }}{4}\left( {2n + 1} \right)^{2} \left( {\frac{a}{b}} \right)^{2} \left( {\frac{{k_{f} }}{{k_{x} }}} \right)\left( {\frac{{k_{x} }}{{a^{2} }}} \right)t}} } + \frac{1}{2}\sum\limits_{{l = 0}}^{\infty } {e^{{ - \frac{{\pi ^{2} }}{{16}}\left[ {\left( {2l + 1} \right)^{2} } \right]t_{D} }} } } \right\}$$

While initial rate is a significant parameter, often we may wish to use initial rate as a normalization parameter, allowing us to see signatures in a scaled fashion. Thus, the real-world values appearing as multipliers in Eqs. 18–20 do not need to be specified if we choose to plot q_j_/q_i_, where the index *j* stands for the number of draining fractures, and the index *i* stands for initial value. Although we can make an interesting analogy with decline curve analysis in plotting the rates, the Bourdet derivative equivalents in average pressure, used in well testing, yield important flow regime signatures.

21 $$\frac{{{\text{q}}_{4} t}}{{\frac{{P_{i} V_{m} }}{\mu }\left( {\frac{{k_{x} }}{{a^{2} }}} \right)}} = \begin{array}{*{20}c} {\left( {\frac{{V_{f} }}{{V_{m} }}} \right)\left( {\frac{{k_{f} }}{{k_{x} }}} \right)\left[ {\frac{{2t}}{{\left( {1 + \frac{b}{a}} \right)^{2} }}} \right]\sum\limits_{{n = 0}}^{\infty } {e^{{ - \frac{{\pi ^{2} }}{4}\left( {2n + 1} \right)^{2} \left[ {\frac{1}{{\left( {1 + \frac{b}{a}} \right)^{2} }}} \right]\left( {\frac{{k_{f} }}{{k_{x} }}} \right)t_{D} }} } } \\ { + \frac{{16{\text{t}}}}{{\pi ^{2} }}\sum\limits_{{l,m = 0}}^{\infty } {\left[ {\left( {2m + 1} \right)^{{ - 2}} + \left( {\frac{{k_{y} }}{{k_{x} }}} \right)\left( {\frac{a}{b}} \right)^{2} \left( {2l + 1} \right)^{{ - 2}} } \right]} {\text{exp}}\left[ { - \frac{{\pi ^{2} }}{4}\left( {\left( {2l + 1} \right)^{2} + \left( {\frac{{k_{y} }}{{k_{x} }}} \right)\left( {\frac{a}{b}} \right)^{2} \left( {2m + 1} \right)^{2} } \right)t_{D} } \right]} \\ \end{array}$$

22 $$\frac{{{\text{q}}_{2} t}}{{\frac{{P_{i} V_{m} }}{\mu }\left( {\frac{{k_{x} }}{{a^{2} }}} \right)}} = 2{\text{t}}\left( {\frac{{V_{f} }}{{V_{m} }}} \right)\left( {\frac{a}{b}} \right)^{2} \left( {\frac{{k_{f} }}{{k_{x} }}} \right)\sum\limits_{{n = 0}}^{\infty } {e^{{ - \frac{{\pi ^{2} }}{4}\left( {2n + 1} \right)^{2} \left( {\frac{a}{b}} \right)^{2} \left( {\frac{{k_{f} }}{{k_{x} }}} \right)t_{D} }} } + t\sum\limits_{{l = 0}}^{\infty } {e^{{ - \frac{{\pi ^{2} }}{4}\left[ {\left( {2l + 1} \right)^{2} } \right]t_{D} }} }$$

and

23 $$\frac{{{\text{q}}_{1} t}}{{\frac{{P_{i} V_{m} }}{\mu }\left( {\frac{{k_{x} }}{{a^{2} }}} \right)}} = 2{\text{t}}\left( {\frac{{V_{f} }}{{V_{m} }}} \right)\left( {\frac{a}{b}} \right)^{2} \left( {\frac{{k_{f} }}{{k_{x} }}} \right)\sum\limits_{{n = 0}}^{\infty } {e^{{ - \frac{{\pi ^{2} }}{4}\left( {2n + 1} \right)^{2} \left( {\frac{a}{b}} \right)^{2} \left( {\frac{{k_{f} }}{{k_{x} }}} \right)t_{D} }} } + \frac{{\text{t}}}{2}\sum\limits_{{l = 0}}^{\infty } {e^{{ - \frac{{\pi ^{2} }}{{16}}\left[ {\left( {2l + 1} \right)^{2} } \right]t_{D} }} }$$

We also see a literal interpretation of the Bourdet derivative from the left-hand side as the cumulative production if the entire production had occurred at the present instantaneous rate.

### Block distribution fracture depletion model development

The single block model as a representative element can be generalized into a heterogeneous model using a block size distribution approach. Irrespective of configurational constraints, we can take a probability distribution with volume-weighted relative contributions. A simple bimodal distribution is most simply demonstrated, but this may be expanded to other common forms used in fracture statistics, e.g. Gaussian and popularized Power-Law or fractal distributions, with the last possible provided maximum and minimum cutoffs are defined.

### Differences from traditional fracture-matrix transfer function shape factor

The development herein captures the transfer of matrix fluid into the adjoining already depleted fracture system. This fluid exchange is classically captured with a fracture-matrix transfer function shape factor, σ, defined by the relation

24 $${\text{q}}=\upsigma \frac{\rho k}{\mu }\left(\overline{P }-{P}_{f}\right).$$

This is recognized as a form of well equation for a finite difference approach. The macroscopic driving force is the difference between average pressure and the pressure at the bounding fracture with all geometry effects lumped into the shape factor. This also makes modelling naturally fractured reservoirs very similar to the extended use of well equations incorporating a shape factor for drainage area shape, non-centered wells, and even well productivity indices.

For spontaneous imbibition, the shape factor was given a generalized physical interpretation as the sum of the ratio of contactable surface, *A*_*j*_, and distance to the center of mass, *d*_*j*_, per unit volume, *V*^25^.

25 $$\upsigma =\frac{1}{V}\sum_{j=1}^{J}\frac{{A}_{j}}{{d}_{j}}.$$

Thus, it carries the dimensions of *L*^*−2*^, an inverse area. Eq. 25, in part, gave a distance over which an macroscopic average driving force, that pulls a portion of fluid characterized by a particular surface-to-volume ratio to a designated capture surface, might act.

The use of a macroscopic average driving force in any Darcy-like expression to relate pressure drop and flowrate, while convenient, overshadows the physics captured in microscopic transport equations and use of local derivatives. While Hazlett and Younis^22^ wanted to see where fluid was being expelled from matrix blocks, hence the interest in the expression for pressure derivative with time at the fracture-matrix interface, here the focus is on overall macroscopic flowrate. Since the depletion process is captured by changes in the average pressure driven by fluid withdrawal and subsequent balancing fluid expansion, an expression equivalent to Eq. 25 is avoided in favour of a relationship between produced fluid volume and the rate of change in average pressure with time. Interestingly, the Green’s function spatial solution is for *P*_*m*_ (*x, y, t*)—*P*_*f*_ , and the averaging process pushes the expression into the form of Eq. 25, though unused as such.

## Results and discussion

**Figure 4:**
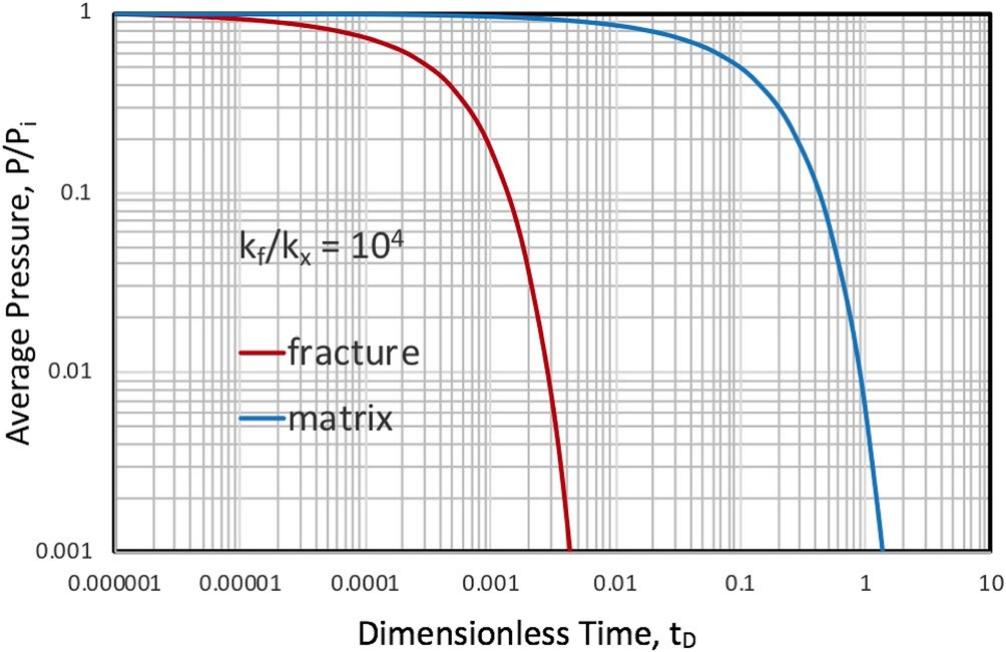
A comparison of fracture versus matrix depletion indicating that for k_f_/k_x_ = 10^4^, the fracture depletion is essentially complete before any appreciable depletion of the matrix. This substantiates computation of drainage in combined systems as independent events. Thus, the matrix boundary condition of constant pressure is reasonable.

Preliminary computations show that provided fracture aperture is on the order of millimeters and matrix permeability 100 mD or less, the depletion of the fracture system will be essentially complete before appreciable expulsion from the matrix, substantiating the use of Eqs. 19–24 to represent production from NFRs. This is supported by Fig. 4 showing the relative pressure depletion in matrix versus fracture for a fracture to matrix permeability ratio of 10^4^.

The alternative would be to solve the complete coupled problem with time dependent boundary conditions for the matrix—a substantially more difficult problem, as advocated by Hassanzadeh and Pooladi-Darvish^26^. Herein, we want to examine a simple model before concluding a more complex one is necessary, especially considering the model is not an exact representation of the geometry and homogeneity in fracture and matrix properties is not expected.

**Figure 5:**
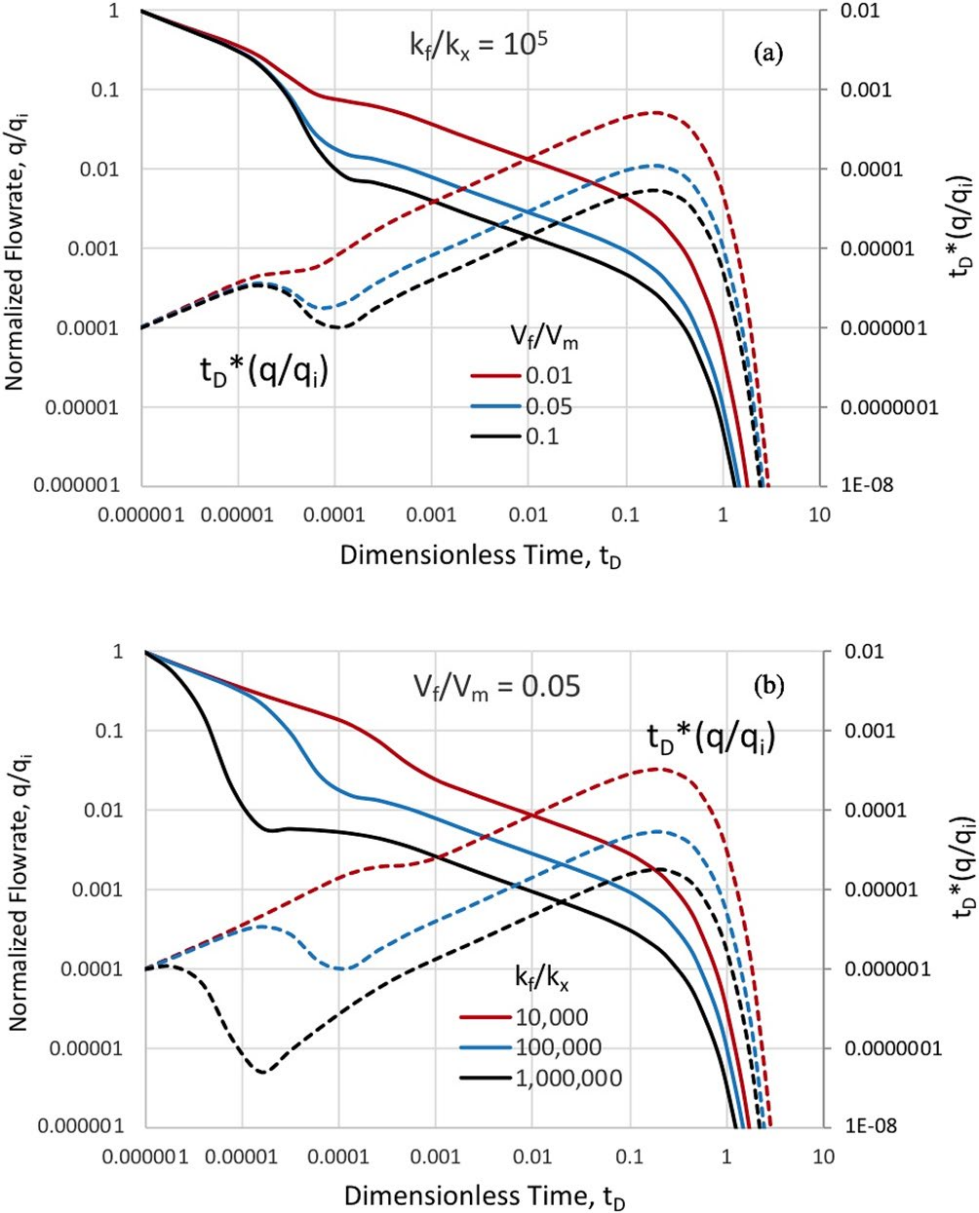
Normalized flowrate and Bourdet derivative for matrix depletion into surrounding fractures: (**a**) Variable fracture-to-matrix volume ratio at constant fracture-to-matrix permeability of 10^5^, and (**b**) Variable fracture-to-matrix permeability at constant fracture-to-matrix volume ratio of 0.05.

Figure 5 shows the interacting systems results for normalized instantaneous rate and the Bourdet derivative equivalent for rate decline with a representative element in the proposed model for naturally fractured reservoirs. This result corresponds to Fig. 1 where the matrix block of unit aspect ratio is drained from both primary and conjugate fracture sets in plan view. In Fig. 5a, we see sensitivity in observed rates as a function of the ratio of fracture-to-matrix volume at a fixed fracture-to-matrix permeability ratio of 10^5^. Figure 5b shows the sensitivity with respect to fracture-to-matrix permeability ratio at fixed volume ratio. The rate of fracture drainage and transition to matrix-dominated flow is dramatically impacted by the permeability ratio. We note that the matrix drainage is 2-D and deviates slightly from a ½ slope, characteristic of a linear flow regime.

**Figure 6:**
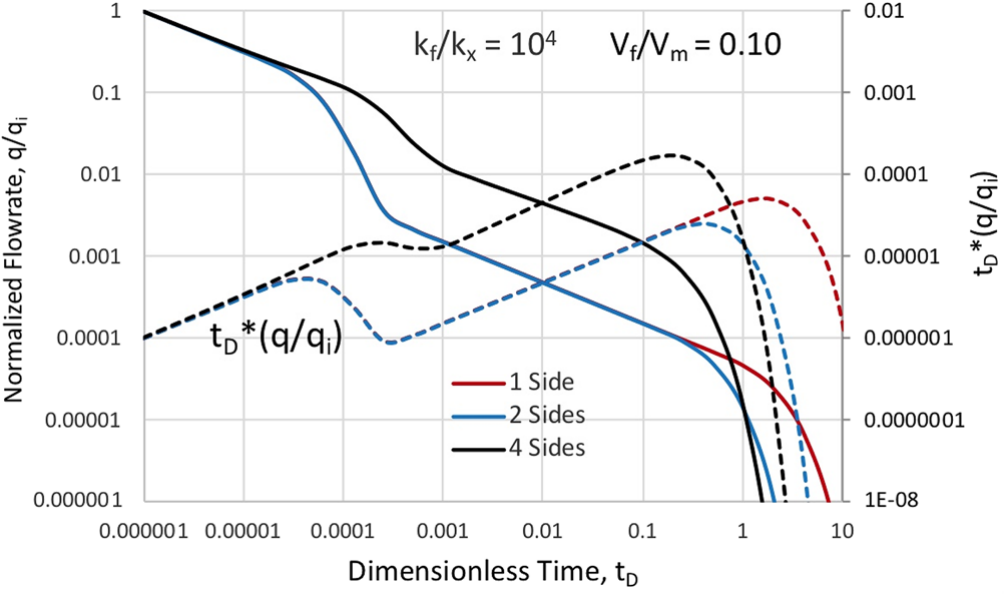
Normalized flowrate versus time for aspect ratio a/b = 1, fixed permeability ratio, k_f_/k_x_ = 10^4^, and the same fracture-to-matrix volume ratio of 0.10 for matrix drained by different numbers of contacting fractures.

Figure 6 illustrates the differences in rate decline due to the choice of model depicted in Figs. 1–3. Some aspects of differences are masked by the normalization to the initial rate. What we see, however is that depletion in the fracture systems is similar, since both the volume and permeability ratios are held constant, and the transition to matrix-dominated flow happens at similar times. However, the matrix block drained by fractures on all sides maintains higher production rates and more rapid system depletion. The differences in model systems drained by only one fracture or a set of parallel fractures are indeed masked by the rate normalization process. Initial rate with two fractures would be twice that of the single fracture. Aside from this rate shift in favor of more fractures, we see the slowest ultimate depletion by the single fracture due to the extra distance required for fluid to traverse the matrix.

**Figure 7:**
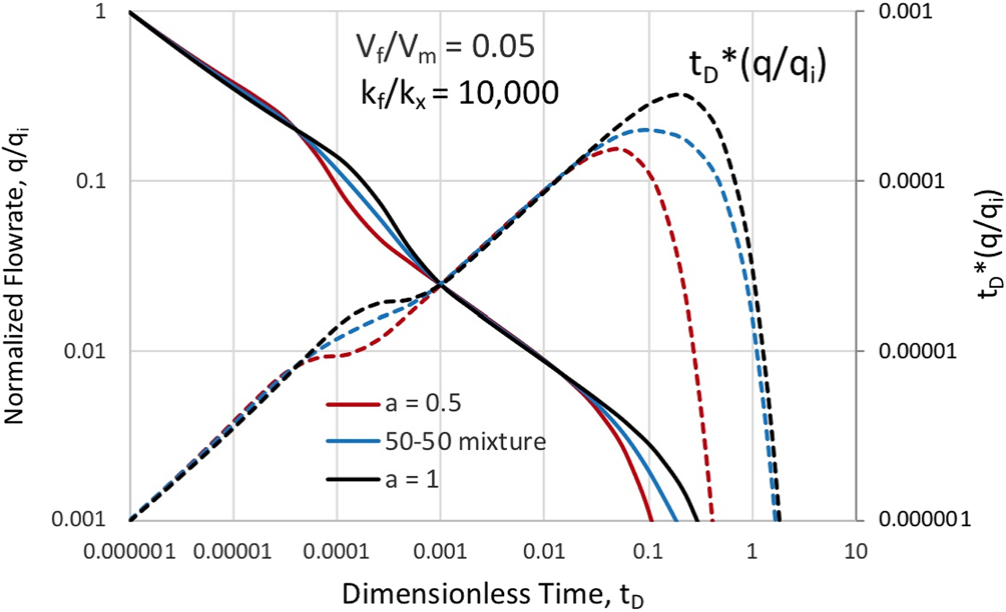
Normalized rate versus time for blocks of different size and a 50–50 mixture for constant aspect ratio a/b = 1 showing similar depletion behavior with a shift in transition and overall depletion time.

Finally, we demonstrate the behavior of a heterogeneous mixture in Fig. 7, where the normalized rate decline and Bourdet derivative equivalent are given for end members with matrix block of identical aspect ratio, *a/b* = 1, but matrix block sizes in a ratio of 2:1. Included is the expected result for a 50–50 mixture of these two block sizes. This difference in depletion times is due to the time stretching process scaling due to the characteristic size seen in *k*_*x*_*/a*^*2*^. More dramatic block size differential would result in greater distinction. While an impact in a specific time range is represented, with the other process variables of volume and permeability ratio undetermined, it may be difficult to detect a heterogeneity signature in the presence of only mild variation. We are reminded that decline curve analysis is an inverse modeling process without a guaranteed unique solution. The difference in matrix block depletion time comes late in the process and may be only useful in identifying the economic limit of reservoir recovery operations. In Fig. 7, we ascertain some ability to discern signatures of mixtures, but with only the composite behaviour observable, the ability to segment distributions is very limited. Average block size and range in block distributions may be somewhat interpretable. Further studies involving well test interpretations instead of simple production data analysis within the context of this model may prove fruitful.

The value in such development depends upon the end use. Signatures and interpretation of naturally fractured reservoirs for reservoir characterization purposes have inherent value^20^. However, there is always danger in interpretation with oversimplified models. The more realism put in the model, the better the understanding; however, there is a recognized limit. As in all inverse problem solving, beyond some number of unknowns, the problem becomes ill-posed. In this light, application of Occam’s razor might be appropriate in that the simplest model that can explain the behaviour and capture it quantitatively is preferred^27^. Certainly, capturing contributions from a distribution with few introduced parameters, such as a fractal dimension^28^, may be worthwhile. On the other hand, there could be immense value in building hybrid numerical models with more embedded analytical constructs. In this manner, many numerical stability problems can be avoided while still being able to entertain more complex physics, such as multiphase flow and PVT.

## Conclusions

New representative element models that include anisotropy were posed and derived for recovery from naturally fractured reservoirs. Analytic solutions to the governing differential equation and constant temperature boundary conditions were taken from Carslaw and Jaeger^23^ and repurposed for the analogous Diffusivity Equation. Average pressure was extracted from such solutions, and since the average pressure is related to cumulative production through material balance, this provides an avenue for new rate decline models for such systems. The analytic derivative of the change in average pressure was related to instantaneous flowrate, and this result multiplied by time was identified as the equivalent of the Bourdet derivative to identify the prevailing flow regime. A physical interpretation was provided for this derivative in relation to the cumulative production as if the current instantaneous rate were constant through all time.

It was determined that fracture and matrix depletion can be treated as independent events provided the permeability ratio of fracture-to-matrix exceeds 10^4^, which is reasonable for most reservoirs with open fractures on the millimetre scale. Thus, we can use the matrix depletion model that assumes the pressure at boundary at t = 0^+^ is constant. After the fracture is depleted, it is assumed that all fluid transferring to the boundary from the matrix is subsequently captured.

The models were tested for sensitivity to the system characterization in terms of volume ratio, *V*_*f*_* /V*_*m*_, and permeability ratio, *k*_*f*_* /k*_*x*_. Results indicate that rate decline transitions in both rate and Bourdet derivative equivalent carry information on these characterizing parameters traditionally sought in well testing. Furthermore, the rate decline signature may carry sufficient information to distinguish between the different representative matrix element models. However, there are other variables, such as block aspect ratio that were not tested and may further complicate the 2-D depletion profile. The square aspect ratio tested carries the most 2-D depressurization character. Other aspect ratios will yield a greater degree of linear flow behaviour and make it more difficult to distinguish between matrix blocks of adverse aspect ratio surrounded by fractures and those drained only by a reduced set, since the matrix depletion in models represented by Figs. 2 and 3 reduces to one-dimensional flow.

Furthermore, the scaling behaviour with respect to characteristic block size dimension, *a*, enables extension of the model to mixtures of block sizes. This is demonstrated with a binary mixture, though more complicated distributions commonly associated with fracture statistics may be entertained. The existence of a readily drainable fracture gathering system would yield the same constant pressure boundary condition for all contributing blocks of varying size and shape. Still, with the non-uniqueness problem in inverse modelling, only model distributions with a limited number of parameters should be entertained, for the averaging process giving rise to a single observed rate may mask our ability to recover heterogeneity characterization.

## Data Availability

The results from equations presented were compiled with approximations to double and triple infinite loops within Excel and can be reproduced by the readers as such.
